# Adhering to dietary guidelines does not yield flavanol intake levels associated with beneficial cardiovascular effects

**DOI:** 10.1039/d6fo00867d

**Published:** 2026-06-08

**Authors:** Javier I. Ottaviani, John W. Erdman, Francene M. Steinberg, JoAnn E. Manson, Howard D. Sesso, Hagen Schroeter, Gunter G. C. Kuhnle

**Affiliations:** a Mars Food and Nutrition, a segment of Mars, Inc. McLean VA 22101 USA; b Department of Food Science and Human Nutrition, University of Illinois at Urbana-Champaign Urbana-Champaign IL USA; c Department of Nutrition, University of California Davis Davis CA 95616 USA; d Division of Preventive Medicine, Department of Medicine, Brigham and Women's Hospital and Harvard Medical School 900 Commonwealth Ave East Boston MA USA; e Department of Epidemiology, Harvard T. H. Chan School of Public Health Boston MA USA; f Department of Food and Nutritional Sciences, University of Reading Whiteknights Reading RG6 6AP UK g.gkuhnle@reading.ac.uk

## Abstract

Outcomes from the COSMOS trial have reinforced the notion of flavanols as important plant-derived bioactives contributing to cardiovascular health. As discussions continue on whether specific dietary reference values for flavanols are warranted, it is possible that existing dietary guidelines emphasizing fruits and vegetables already yield sufficient flavanol intake levels. If this were the case, developing flavanol specific dietary reference values might be unnecessary. This study therefore aimed at assessing whether adherence to dietary recommendations for fruit and vegetable intake and overall diet quality achieves flavanol intake levels of 500 mg day^−1^, the amount proven to mediate cardiovascular benefits in the COSMOS trial. Flavanol intake was objectively evaluated using two validated and complementary biomarkers, 5-(3′,4′-dihydroxyphenyl)-γ-valerolactone metabolites (gVLM_B_) and structurally related (−)-epicatechin metabolites (SREM_B_), in two geographically distinct studies: COSMOS (US; *n* = 6509) and EPIC-Norfolk (UK; *n* = 24 154). The results showed that higher fruit and vegetable intakes and diet quality (assessed *via* the alternative healthy eating index-aHEI) were associated with increased flavanol intake in COSMOS. Nevertheless, fewer than 25% of participants meeting dietary guidelines achieved an estimated flavanol intake of ≥500 mg day^−1^. Similar findings were observed in EPIC-Norfolk as well as through flavanol intake simulations considering fruits and vegetables commonly consumed in the US diet. In conclusion, adherence to existing dietary guidelines does not yield flavanol intake levels comparable to those shown to provide cardiovascular benefits in COSMOS. Thus, specific dietary reference values for flavanols may still be necessary if aiming to increase the intake of these dietary compounds.

## Abstract 2

Adherence to dietary guidelines does not yield flavanol intakes comparable to those shown to provide cardiovascular benefits in COSMOS. Dietary reference values for flavanols may be necessary to increase the intake of these dietary compounds.

## Graphical abstract

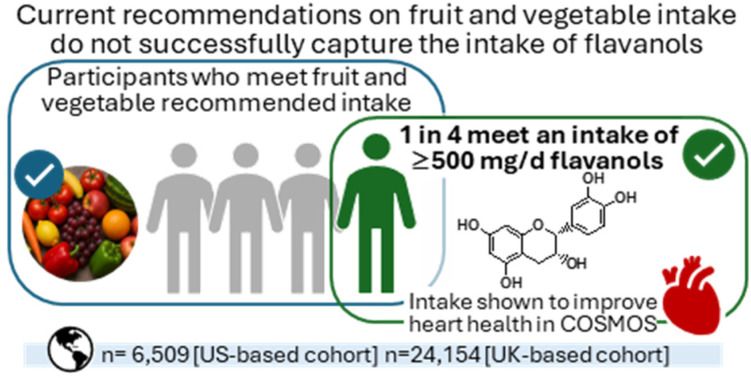

## Introduction

Flavanols are a distinct group of plant-derived polyphenolic bioactives.^1,2^ Evidence from the COSMOS trial, the largest randomized controlled study on polyphenols to date, demonstrated that an intake of 500 mg day^−1^ of flavanols significantly reduced cardiovascular disease (CVD) mortality by 27% in intention-to-treat analyses, total cardiovascular events by 15% in per-protocol analyses and major cardiovascular events by 16% as a *post-hoc* endpoint analysed in intention-to-treat analyses in healthy older adults.^3^ These findings have strengthened the view of flavanols as important dietary compounds for cardiovascular health, which have fuelled ongoing debates on whether specific dietary recommendations for flavanols and other bioactives should be established.^4–7^ Recently, an expert scientific panel commissioned by the Academy of Nutrition and Dietetics in the United States proposed an intake of 400–600 mg day^−1^ for cardiometabolic health.^8^ However, official dietary recommendations for flavanols have not yet been considered. Because fruits and vegetables, including legumes, are major sources of flavanols, and fruits and vegetables are emphasized in current dietary guidelines in the US,^9^ UK^10^ and by the World Health Organization,^11^ it is plausible that adherence to these dietary guidelines could already deliver sufficient flavanol intake levels. Yet no data currently demonstrate whether following recommended intakes of fruits, vegetables, and overall healthy dietary patterns achieves flavanol levels associated with the cardiovascular benefits as shown in COSMOS trial. Addressing this point will provide valuable insights to determine the necessity of developing specific dietary recommendations for flavanols.

**Fig. 1:**
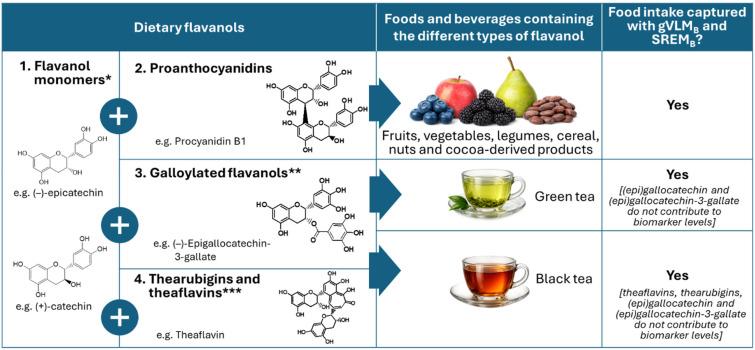
Main group of dietary flavanols present in different flavanol-containing food and beverages in the diet and contribution to flavanol biomarkers. *(−)-Epicatechin is the only flavanol monomer that contributes to SREM_B_ levels; ** (Epi)catechin-3-gallates are the galloylated flavanols that give rise to gVLM_B_ levels after intake, but not (*epi*)gallogatechins and (*epi*)gallogatechin-3-gallates; ***Thearubigins and theaflavins are exclusively present in black tea and the intake of these compounds does not give rise to gVLM_B_ and SREM_B_ levels. gVLM_B_: 5-(3′,4′-dihydroxyphenyl)-γ-valerolactone metabolites; SREM_B_: structurally related (−)-epicatechin metabolites.

Flavanols are found in fruits like pome fruits, berries and stone fruit, vegetables like pinto, kidney and fava beans as well as other products like tea and cocoa-derived products. Previous studies have relied on self-reported dietary assessments to assess flavanol intake. However, these tools have shown significant limitations.^12,13^ In contrast, validated nutritional biomarkers, such as 5-(3′,4′-dihydroxyphenyl)-γ-valerolactone metabolites (gVLM_B_) and structurally related (−)-epicatechin metabolites (SREM_B_), offer objective estimates of flavanol intake.^14,15^ Furthermore, gVLM_B_ and SREM_B_ can assess the intake of those types of flavanols present in fruits and vegetables as well as those tested in the COSMOS trial (Fig. 1). Comprehensive dietary and biomarker data are also available for two large studies, COSMOS (USA) and EPIC Norfolk (UK), making these cohorts ideally suited to examine the link between dietary recommendations and flavanol intake.

Here, we tested whether adhering to recommended levels of fruit and vegetable intake and an overall healthy dietary pattern results in flavanol intakes of at least 500 mg day^−1^. This threshold reflects the intake shown to confer benefits in the COSMOS trial^3^ and also represents the average intake recommended by the guidelines commissioned by the Academy of Nutrition and Dietetics.^8^ We used more recently collected data and assays in COSMOS to develop and assess this hypothesis and data from EPIC Norfolk to assess replication in an independent, population-based sample with differing dietary patterns and temporal context.

## Methodology

### Study design

#### COSMOS

The COSMOS trial (NCT02422745) is a randomized, double-blind, placebo-controlled, 2-by-2 factorial trial of a daily cocoa extract as a source of flavanols and a daily multivitamin for prevention of respective cardiovascular disease (CVD) and cancer among 21 442 US adults (12 666 women aged ≥65 years and 8776 men aged ≥60 years), free of major CVD and recently diagnosed cancer. COSMOS recruited participants from three main sources, including the Women's Health Imitative (WHI), non-randomized recruitment respondents for the VITamin D and OmegA-3 TriaL (NCT01169259), and those identified through mass mailings and media efforts. Contact was made with nearly three million prospective participants. There were 21 442 participants ultimately enrolled and randomized into COSMOS. For the aspects relevant to this study, we conducted a *post hoc* analysis of data collected before randomization into COSMOS (*i.e.* before participants received any of the interventions). Fruit and vegetable intake as well as diet quality assessments were derived from a semi-quantitative food frequency questionnaire that participants completed during the run-in phase of the study.^16,17^ In addition, a subgroup of participants (*n* = 6509) provided spot urine samples also during the run-in phase of the study in which flavanol biomarkers were measured. Non-fasting spot urine samples were collected as part of clinic visits for health checks as described previously.^3^ Further details of the protocol and main findings of the study were previously published.^3^ All participants provided written informed consent, and study approvals were obtained by the Institutional Review Board (IRB) at Mass General Brigham.

#### EPIC Norfolk

The European Prospective Investigation into Cancer and Nutrition (EPIC)-Norfolk is an observational cohort study in Norfolk, UK. Between 1993 and 1997, 30 447 women and men aged between 40 and 79 years were recruited for the Norfolk cohort of the EPIC study, and 25 639 attended a health examination.^18^ Diet was assessed by 7 day diary (7DD), whereby the first day of the diary was completed as a 24 h recall (24HDR) with a trained interviewer and the remainder completed during subsequent days. Diary data were entered, checked, and calculated using the in-house dietary assessment software DINER (Data into Nutrients for Epidemiological Research) and DINERMO.^19^ Non-fasting urine samples were collected during the health examination and stored at −20 °C until analysis in which flavanol biomarkers were measured.^20^ In addition, non-fasting blood samples were taken by venipuncture and stored in serum tubes in liquid nitrogen. Plasma vitamin C was measured in a subset of participants (*n* = 21 177) using a fluorometric assay as described previously.^21^ The study was approved by the Norwich Local Research Ethics Committee, all participants gave written, informed consent, and all methods were carried out in accordance with relevant guidelines and regulations.

### Flavanol biomarkers

Flavanol biomarkers assessed in this study included the urinary levels of 5-(3′,4′-dihydroxyphenyl)-γ-valerolactone metabolites (gVLM_B_) and of the structurally related (−)-epicatechin metabolites (SREM_B_).^14,15^ While gVLM_B_ informs on the intake of flavanols in general (including (*epi*)epicatechin, (*epi*)catechin-3-gallate, and procyanidins), SREM is a specific biomarker of the intake of (−)-epicatechin, one of the main bioactive flavanol compounds and present within the 500 mg day^−1^ of flavanols tested in COSMOS. As such, gVLM_B_ and SREM_B_ provide information on the intake of the cocoa flavanols tested in COSMOS as well as the flavanols present in fruits and vegetables (Fig. 1). While gVLM_B_ and SREM_B_ also provides information on the intake of flavanols from tea, one of the main dietary sources of flavanols, these biomarkers reflect the intake of those flavanols in tea that are also found in fruits and vegetables (Fig. 1). Flavanols that are specific to tea, specially theaflavins and thearubigins derived from black tea processing, and absent in fruits and vegetables, do not influence gVLM_B_ and SREM_B_ levels.^14,15^ Currently, there are no validated biomarkers to assess the intake of tea-specific flavanols, such as (*epi*)gallocatechins, theaflavins and thearubigins.

SREM_B_ and gVLM_B_ have different systemic half-lives (estimated in 2 h and 6 h after the intake of (−)-epicatechin, respectively^22^), thus a combination of both biomarkers allows capturing different periods after flavanol intake. Adherence with flavanol intake consistent with the intake of 500 mg day^−1^ was estimated by using a combination of SREM_B_ and gVLM_B_ concentrations as described previously.^23^ In brief, we calculated threshold values for the concentration of SREM_B_ and gVLM_B_ that could be expected after the intake of 500 mg of flavanols from a dose-escalation study that was part of the validation of SREM_B_ and gVLM_B_ as biomarkers.^14,15^ In this manner, participants with SREM_B_ urinary concentration above 7.77 µM or a gVLM_B_ urinary concentration above 18.21 µM were considered to have an intake of flavanols of at least 500 mg day^−1^. These thresholds were defined as the lower 95% CI limit of the expected concentration of gVLM_B_ and SREM_B_ after the intake of 500 mg of flavanols,^23^ which results in an overestimation of the proportion of participants meeting an intake of flavanols of at least 500 mg day^−1^ and thus introduces a bias in favour of the null-hypothesis (*i.e.* adhering to dietary recommendations results in higher flavanol intake). To assess robustness of this approach, a sensitivity analysis was conducted to assess how changing the thresholds selected for flavanol biomarkers affect these outcomes (SI Fig. S2). The analysis showed that decreasing the thresholds of both flavanol biomarkers resulted in an increase the proportion of participants meeting a 500 mg of flavanols. However, even a reduction of both thresholds by 50% still resulted in less than 50% of participants meeting an intake of 500 mg of flavanols among participants meeting recommendations for fruit and vegetable intake.

gVLM_B_ corresponded to the sum of 5-(4′-hydroxyphenyl)-γ-valerolactone-3′-sulfate and 5-(4′-hydroxyphenyl)-γ-valerolactone-3′-glucuronide meanwhile SREM_B_ corresponded to the sum of (−)-epicatechin-3′-glucuronide, (−)-epicatechin-3′-sulafte and 3′-*O*-methyl-(−)-epicatechin-5-sulfate. These metabolites were quantified using validated LC-MS methods using authentic and isotopically labelled standards.^20^ Unadjusted biomarker concentrations were used as adjustment by creatinine is known to introduce bias^24^ and adjusting by specific gravity did not materially change ranking of participants as previous shown.^23^

### Diet quality assessment

#### Intake of fruit and vegetable

For COSMOS, we determined total fruit and vegetable intake (as servings per day) using data from a semi-quantitative food frequency questionnaire (https://www.cosmostrial.org/investigators/MAIN%20COSMOS%20FFQ_53683.pdf) completed by participants during the run-in phase of the study prior to randomization.^16,17^ For EPIC Norfolk, we determined total fruit and vegetable intake (as g day^−1^) from 7 day food diaries.^19^ In addition, vitamin C in plasma was assessed as a biomarker of fruit and vegetable intake.^25^ Given the importance of tea as a dietary source of flavanols, tea intake was also assessed in both COSMOS and EPIC Norfolk, using the same corresponding dietary assessment methods for fruit and vegetable intake.

#### Diet quality

Diet quality was assessed with the alternative healthy eating index (aHEI). aHEI is an alternative index to the Healthy Eating Index that reflects earlier iterations of the Dietary Guidelines for Americans, including a modification to better predict impact on chronic diseases, including CVD.^26^ In addition to total fruit and total vegetable intake, aHEI also included the assessment of nuts, legumes and cereal fibre, that could represent additional sources of flavanol intake.^27,28^ aHEI was calculated for COSMOS but not for EPIC-Norfolk. Diet scores range from 0 to 87.5, in which 87.5 indicates perfect adherence. Diet quality in EPIC-Norfolk was assessed with a modified version of the Eatwell Guide score^29^ that included eight food and nutrient groups: fruit and vegetables, oily fish, other fish, red and processed meat, total fibre, total salt, saturated fatty acids and total fat. The modified Eatwell Guide score ranges from 0 to 8, and participants were grouped into four categories of adherence based on the number of dietary recommendations met: very low adherence (score 0–2), low adherence (score 3–4) and intermediate adherence (score 5–6) and high adherence score (7–8). The score reflects adherence to current dietary recommendations in England.

### Simulation of flavanol intake from fruit and vegetable consumption

Flavanol intake from fruit and vegetable consumption was simulated using food composition data from Phenol-Explorer (PhenolExplorer) and 2017 NHANES data on fruit and vegetable consumption patterns. We evaluated varying numbers of daily portions (using sizes based on 21 CFR § 101.12) and different food selection strategies: (1) random (unweighted), (2) weighted by US consumption frequency (NHANES 2017), and (3) weighted by flavanol content. For each combination of portion number, selection strategy, and sampling scheme (with or without replacement), we conducted 10 000 Monte Carlo simulations. At each iteration, flavanol content of a selected food item was drawn from a uniform distribution defined by the reported minimum and maximum concentrations in Phenol-Explorer, based on the sum of individual flavanols that give rise to flavanol biomarkers, including (−)-epicatechin, (+)-catechin, epicatechin 3-*O*-gallate, catechin 3-*O*-gallate, and procyanidins from dimers up to decamers (Fig. 1). The simulations used Full data, and code can be found here: https://gitlab.act.reading.ac.uk/xb901875/fruit_vegetable_flavanols.

### Statistical analysis

All analyses were conducted with R version 4.3 in RStudio 2024.09.0. Tables were created using the table one package,^30^ regression analyses using the rms package^31^ and graphics using ggplot2.^32^ Dietary data were log 2 transformed before analysis. LRM was used to conduct logistic regressions with “meeting a biomarker-estimated flavanol intake of at least 500 mg day^−1^” as dependent variable. Models were adjusted by age and sex, using restricted cubic splines for all continuous variables. Fruit and vegetable intakes were divided into quartiles using the cut function. For the aHEI score, ties prevented even distribution into quartiles. We added minimal random jitter (±0.0001) to break ties, repeating this process 1000 times and averaging results across iterations to avoid dependence on any single arbitrary tie-breaking assignment. For the Eatwell Guide score, participants were not divided into quartiles but grouped into four different categories based on score as described above. Missing values were excluded from the analysis.^32^

## Results

### Study population

Biomarker data were available for *n* = 6509 COSMOS participants and *n* = 24 154 EPIC Norfolk participants (Table 1). In general, participants in COSMOS were older, had a slightly higher proportion of males, and a higher BMI compared to participants in EPIC Norfolk; EPIC Norfolk was less ethnically diverse. As intake of food items, including fruits and vegetables, was reported in different units in COSMOS (*i.e.* servings per day) and EPIC Norfolk (*i.e.* g day^−1^), values were not directly compared between cohorts except for tea, which was consumed at much higher levels in EPIC Norfolk (773 ± 530 g day^−1^) than COSMOS (134 ± 261 g day^−1^). Concentrations of gVLM_B_, the more general biomarker of flavanol intake, were not different between studies (Table 1 and SI Fig. S1). However, SREM_B_ levels, the specific biomarker of (−)-epicatechin intake, were higher in participants in EPIC Norfolk compared to those in COSMOS (Table 1 and SI Fig. S1). Data from both biomarkers were combined to estimate the proportion of participants meeting an intake of at least 500 mg day^−1^ of flavanols.^23^ The percentage of the population that met the biomarker-estimated flavanol intake of at least 500 mg day^−1^ was 19.2% and 17.9% in COSMOS and EPIC, respectively (Table 1). In both studies, men were more likely to meet a flavanol intake of at least 500 mg day^−1^ (OR 1.36 (95% CI 1.29; 1.45); adjusted by age, BMI and recruitment cohort). In contrast, older participants of COSMOS were more likely to meet a flavanol intake of at least 500 mg day^−1^ (OR 1.21 (1.06; 1.39); 65 *vs.* 75 years; adjusted by sex and BMI), while this was reversed in EPIC Norfolk (OR 0.91 (0.83; 0.99) 65 *vs.* 75 years; adjusted by sex and BMI). Likewise, normal-weight participants of COSMOS were more likely to meet a flavanol intake of at least 500 mg day^−1^ (OR 0.92 (0.85; 0.98)) compared with obese participants, while the opposite was the case in EPIC (OR 1.12 (1.07; 1.17); SI Table S1).

**Table 1:** Characteristics of study population in COSMOS (US) and EPIC Norfolk (UK). Data are expressed as mean ± SD, unless otherwise stated

	COSMOS	EPIC Norfolk	*p* a
Participants (*n*)	6 509	24 154	
Age (years)	71.0 ± 6.3	58.6 ± 9.3	<0.001
Male (*n*)	3178 (48.8%)	10 879 (45.0%)	<0.001
BMI (kg m^−2^)	27.6 ± 5.3	26.3 ± 3.9	<0.001
Fruit & vegetable intake (COSMOS, servings per day; EPIC Norfolk, g day^−1^)^b^	5.71 ± 4.13	282 ± 169	nd
Fruit intake (COSMOS, servings per day; EPIC Norfolk, g day^−1^)^c^	2.27 ± 1.96	161 ± 133	nd
Vegetable intake (COSMOS, servings per day; EPIC Norfolk, g day^−1^)^b^	3.45 ± 2.82	121 ± 74	nd
Tea intake (g day^−1^)^b^	134 ± 261	773 ± 530	<0.001
Plasma vitamin C (µmol L^−1^)	nd^c^	53.5 ± 20.4	nd
aHEI score	42.56 ± 10.82	nd^c^	nd
Eatwell Guide score^b^	nd	4 [3; 4]	nd
gVLM_B_ (µmol L^−1^)^b^	3.30 [0.78, 11.00]	3.24 [0.83, 10.47]	0.126
SREM_B_ (µmol L^−1^)^b^	0.47 [0.10, 1.88]	0.87 [0.23, 2.38]	<0.001
Participants meeting a biomarker-estimated flavanol intake of at least 500 mg day^−1^ (*n*)	1248 (19.2%)	4317 (17.9%)	0.016

a Test for difference is between the two cohorts determined by *t*-test, except for gVLM_B_ and SREM_B_ that were compared using Fischer's test.

b Data are expressed as median and IQR.

c Not determined. gVLM_B_: 5-(3′,4′-dihydroxyphenyl)-γ-valerolactone metabolites; SREM_B_: structurally related (−)-epicatechin metabolites.

### Association between diet quality and biomarker-estimated flavanol intake in COSMOS

In order to investigate whether participants that adhere to current dietary guidelines have a higher flavanol intake, we divided participants into quartiles based on fruit and vegetable intake and diet quality assessed as aHEI score (Table 2). Participants in the top quartile of fruit and vegetable intake met and often surpassed the 5 servings per day recommendation by the Dietary Guidelines for Americans.^9^ Similarly, participants in the top quartile of aHEI showed scores between 58% to 93% of maximal score, suggesting that participants in the highest quartile of aHEI presented high adherence to a healthy dietary pattern. However, despite meeting these recommendations and following a healthy dietary pattern, only 21% of participants consumed at least 500 mg day^−1^ of flavanols (Table 2). These figures were not substantially higher than the 19.2% of participants with a flavanol intake of at least 500 mg day^−1^ determined for the entire COSMOS cohort (Table 1). As tea can be an important source of flavanols,^33^ we also included tea in our analyses. COSMOS participants reported relatively low tea consumption, with over one-third reporting no intake. There was no significant difference in the proportion of participants meeting the 500 mg day^−1^ flavanol threshold between high and low tea consumers (Table 2).

**Table 2:** Number of COSMOS^b^ participants meeting a biomarker-estimated flavanol intake of at least 500 mg day^−1^ across different quartiles of fruit and vegetable intake, tea intake and diet quality. Data are shown as mean ± SD

Dietary assessment	Quartile 1	Quartile 2	Quartile 3	Quartile 4	*p* a
**Fruit & vegetable intake (serving per day)**	**0.1–3.4**	**3.5–5**	**5–7.1**	**7.1–121** e	
COSMOS participants (*n*)	1549	1547	1548	1548	
Age (years)	69.8 ± 5.9	70.5 ± 5.9	71.6 ± 6.4	72.2 ± 6.7	<0.001
Male (*n*)	831 (54%)	770 (50%)	704 (46%)	632 (41%)	<0.001
Participants meeting a biomarker-estimated flavanol intake of at least 500 mg day^−1^ (*n*)	265 (17%)	298 (19%)	297 (19%)	322 (21%)	0.075
**Fruit intake (serving per day)**	**0–1.1**	**1.1–1.9**	**1.9–2.9**	**2.9–36.1** e	
COSMOS participants (*n*)	1554	1553	1539	1548	
Age (years)	69.7 ± 5.7	70.7 ± 6.1	71.4 ± 6.2	72.32 ± 6.9	<0.001
Male (*n*)	818 (53%)	740 (48%)	707 (46%)	674 (44%)	<0.001
Participants meeting a biomarker-estimated flavanol intake of at least 500 mg day^−1^ (*n*)	267 (17%)	301 (19%)	308 (20%)	306 (20%)	0.167
**Vegetable intake (serving per day)**	**0–1.9**	**1.9–2.9**	**2.9–4.3**	**4.3–85.1** e	
COSMOS participants (*n*)	1553	1555	1550	1552	
Age (years)	70.2 ± 6.2	70.4 ± 6.0	71.6 ± 6.4	71.9 ± 6.4	<0.001
Male (*n*)	845 (54%)	800 (51%)	684 (44%)	621 (40%)	<0.001
Participants meeting a biomarker-estimated flavanol intake of at least 500 mg day^−1^ (*n*)	287 (19%)	300 (19%)	297 (19%)	305 (20%)	0.868
**Tea intake (servings per day)** c	**0–0.1 (bottom half)**	**0.1–14 (top half)**			
COSMOS participants (*n*)	3433	2732			
Age (years)	70.8 ± 6.4	71.3 ± 6.1	0.001		
Male (*n*)	1872 (55%)	1054 (39%)	<0.001		
Participants meeting a biomarker-estimated flavanol intake of at least 500 mg day^−1^ (*n*)	651 (20%)	530 (20%)	0.689		
**aHEI** d **score**	**12.5–34.5**	**34.5–42.5**	**42.5–50.5**	**50.5–81.5**	
COSMOS participants (*n*)	1481	1480	1480	1480	
Age (years)	70.2 ± 6.1	71.0 ± 6.4	71.2 ± 6.3	71.7 ± 6.4	<0.001
Male (*n*)	793 (54%)	756 (51%)	756 (47%)	696 (38%)	<0.001
Participants meeting a biomarker-estimated flavanol intake of at least 500 mg day^−1^ (*n*)	240 (16%)	281 (19%)	281 (19%)	283 (22%)	<0.001

a Test for diff**e**rence is between quartiles was determined by ANOVA except for aHEI that was determined using a bootstrapping approach with meta-regression of results. Differences were determined using meta-regression analyses.

b COocoa supplement and multivitamin outcomes study.

c Almost 50% of COSMOS participants did not consume any tea and a division into quartiles was therefore not possible and data presented correspond to top and bottom half of the population.

d aHEI: alternative healthy eating Index.

e Fewer than 1% of participants reported unrealistically high fruit and vegetable intake (99th percentile: 8, 11 and 18 servings per day of fruits, vegetables and fruit and vegetables).

We initially compared unadjusted quartiles of fruit, vegetable, fruit and vegetable intake, and aHEI score in relation to meeting the flavanol intake threshold of 500 mg day^−1^. To investigate whether these association held after accounting for potential confounders, we calculated odds ratios (OR) adjusted for age, sex, and BMI. Participants in the highest aHEI quartile had 26% higher odds of meeting the 500 mg day^−1^ threshold compared to those in the lowest quartile (Table 3). For all other markers of healthy diet, no significant associations were observed (Table 3). Additional analyses showed that fruit intake, aHEI and tea intake were weakly but significantly associated with SREM_B_ and gVLM_B_ concentrations (SI Table S2).

**Table 3:** Association between food intake and diet quality score and meeting a biomarker-estimated flavanol intake of at least 500 mg day^−1^ in COSMOS. Odds ratio (OR) comparing top *versus* bottom quartiles of diet quality score among COSMOS participants. Data was log 2 transformed and regression analysis was adjusted for age, sex, and BMI

Dietary assessment	OR (95% CI)	*p*
Fruit & vegetable intake	1.10 (1.01; 1.19)	0.06
Vegetable^a^ intake	1.04 (0.95; 1.14)	0.44
Fruit^a^ intake	1.05 (0.96; 1.16)	0.22
Tea intake	0.99 (0.93; 1.06)	0.30
aHEI score	1.26 (1.14; 1.39)	<0.001

a Model additionally adjusted for fruit and vegetable intake respectively. *p*-Value for Wald test.

### Association between diet quality and biomarker-estimated flavanol intake in EPIC-Norfolk

In order to investigate whether the findings obtained in COSMOS participants (Tables 2 and 3) are also observed in a different cohort and of a different geographical region, the association between fruit and vegetable intake and odds to meet a flavanol intake of at least 500 mg day^−1^ was assessed in participants of EPIC Norfolk (Table 4). Participants in the top quartile of fruit and vegetable intake indeed showed intakes meeting or surpassing the amounts recommended by the 5-a-day recommendation.^9,10^ Instead of using aHEI, which was not available, we used plasma vitamin C as surrogate marker of a healthy diet as described previously.^20^ Only 16% of participants in the top quartile presented a flavanol intake of at least 500 mg day^−1^ (Table 4). Similar to COSMOS, adhering to a higher fruit and vegetable intake did not result in higher odds to meet a flavanol intake of at least 500 mg day^−1^ (Table 4). Similar findings were observed when comparing number of participants meeting a flavanol intake of at least 500 mg day^−1^ between top and bottom quartiles of fruit intake and vegetable intake (Table 4). Differences in the number of participants meeting a flavanol intake of at least 500 mg day^−1^ were more pronounced between top and bottom quartiles of vitamin C levels (Table 4). Consistently, there was a modest inverse relationship between fruit and vegetable intake and the odds of meeting a flavanol intake of at least 500 mg day^−1^ in EPIC Norfolk participants (Table 5). Similar findings were observed when assessing fruit intake, vegetable intake, the Eatwell Guide score and plasma vitamin C (Table 5). Indeed, participants with the best adherence to the UK dietary guidelines were least likely to consume at least 500 mg day^−1^ of flavanols. These results show that those not following dietary recommendations generally were more likely to have a flavanol intake of 500 mg day^−1^ as confirmed by the adjusted ORs (Table 5). In contrast, those with high tea intake were more likely to attain 500 mg day^−1^ of flavanol intake, albeit even in the highest quartile of tea intake, only 19% of participant achieved this intake (Table 5).

**Table 4:** Number of EPIC Norfolk participants meeting a biomarker-estimated flavanol intake of at least 500 mg day^−1^ across different quartiles of fruit and vegetable intake, tea intake and vitamin C concentration. Data are shown as mean ± SD

Dietary assessment	Quartile 1	Quartile 2	Quartile 3	Quartile 4	*p* a
**Fruit & vegetable intake (g day** ^**−1**^ **)**	0–163.8	163.8–255.8	255.8–368.9	368.9–2375	
EPIC-Norfolk participants (*n*)	6010	6009	6009	6010	
Age (years)	57.74 (9.54)	58.62 (9.36)	59.02 (9.21)	58.88 (8.85)	<0.001
Male (*n*)	3192 (53.1)	2773 (46.1)	2509 (41.8)	2343 (39.0)	<0.001
Participants meeting a biomarker-estimated flavanol intake of at least 500 mg day^−1^ (*n*)	1205 (20.0)	1093 (18.2)	1036 (17.2)	959 (16.0)	<0.001
**Fruit intake (g day** ^**−1**^ **)**	0–63.4	63.4–136.6	136.6–226.6	226.6–2100	
EPIC-Norfolk participants (*n*)	6010	6009	6009	6010	
Age (years)	57.62 (9.41)	58.54 (9.36)	59.17 (9.18)	58.93 (8.99)	<0.001
Male (*n*)	3358 (55.9)	2688 (44.7)	2500 (41.6)	2271 (37.8)	<0.001
Participants meeting a biomarker-estimated flavanol intake of at least 500 mg day^−1^ (*n*)	1195 (19.9)	1086 (18.1)	1032 (17.2)	980 (16.3)	<0.001
**Vegetable intake (g day** ^**−1**^ **)**	0–72.2	72.2–109.7	109.7–157	157–1180.8	
EPIC-Norfolk participants (*n*)	6010	6009	6009	6010	
Age (years)	58.11 (9.67)	58.88 (9.24)	58.64 (9.19)	58.62 (8.91)	<0.001
Male (*n*)	2847 (47.4)	2712 (45.1)	2619 (43.6)	2639 (43.9)	<0.001
Participants meeting a biomarker-estimated flavanol intake of at least 500 mg day^−1^ (*n*)	1165 (19.4)	1084 (18.0)	1053 (17.5)	991 (16.5)	<0.001
**Tea intake (g day** ^**−1**^ **)**	0–417	418–731	731–1069	1069–6975	
EPIC-Norfolk participants (*n*)	6016	6007	6006	6009	
Age (years)	56.74 (8.99)	59.61 (9.32)	59.84 (9.29)	58.08 (9.09)	<0.001
Male (*n*)	2694 (44.8)	2552 (42.5)	2599 (43.3)	2972 (49.5)	<0.001
Participants meeting a biomarker-estimated flavanol intake of at least 500 mg day^−1^ (*n*)	899 (14.9)	1084 (18.0)	1161 (19.3)	1149 (19.1)	<0.001
**Eatwell Guide score**	0–2	3–4	5–6	7–8	
EPIC-Norfolk participants (*n*)	4914	13 612	5247	265	
Age (years)	56.77 (9.34)	58.60 (9.30)	60.08 (8.79)	59.67 (8.70)	<0.001
Male (*n*)	2561 (52.1)	5753 (42.3)	2360 (45.0)	143 (54%)	<0.001
Participants meeting a biomarker-estimated flavanol intake of at least 500 mg day^−1^ (*n*)	999 (20.3)	2440 (17.9)	826 (15.7)	28 (10.6)	<0.001
**Vitamin C (µmol L** ^**−1**^ **)**	3.0–41	41.9–54	54.1–66	66.5–242	
EPIC-Norfolk participants (*n*)	5474	5294	5324	5085	
Age (years)	59.55 (9.36)	58.43 (9.34)	57.88 (9.03)	58.02 (9.08)	<0.001
Male (*n*)	3476 (63.5)	2799 (52.9)	2048 (38.5)	1295 (25.5)	<0.001
Participants meeting a biomarker-estimated flavanol intake of at least 500 mg day^−1^ (*n*)	1105 (20.2)	969 (18.3)	934 (17.5)	807 (15.9)	<0.001

a Test for difference is between quartiles was determined by ANOVA.

**Table 5:** Association between food intake and diet quality score and meeting a biomarker-estimated flavanol intake of at least 500 mg day^−1^ in EPIC Norfolk. Odds ratio (OR) comparing top *versus* bottom quartiles of diet quality score among EPIC Norfolk participants. Data was log 2 transformed and regression analysis was adjusted for age, sex, and BMI

Dietary assessment	OR (95% CI)	*p*
Fruit & vegetable intake	0.91 (0.87; 0.95)	<0.001
Vegetable^a^ intake	0.99 (0.98; 1.00)	0.006
Fruit^a^ intake	1.00 (0.99; 1.01)	0.005
Tea intake	1.14 (1.09; 1.20)	<0.001
Eatwell Guide score	0.94 (0.91; 0.97)	<0.001
Vitamin C	0.92 (0.88; 0.97)	0.003

a Model additionally adjusted for fruit and vegetable intake respectively. *p*-Value for Wald test.

Further analyses investigating the association between diet quality flavanol biomarkers were conducted (SI Table S3). Intake of tea also showed slightly stronger (0.1 < *β* < 0.3) associations with both flavanol biomarkers, SREM_B_ and gVLM_B_ (SI Table S3).

### Fruit and vegetable intake and flavanol intake simulation

**Fig. 2:**
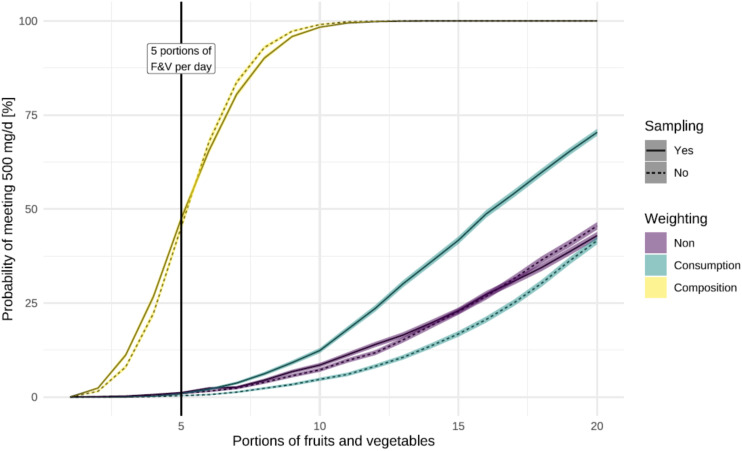
Probability of consuming at least 500 mg day^−1^ of flavanols with different numbers of servings of fruits, vegetables (including legumes) and nuts (F&V) using different selection methods, including weighing and sampling. F&V selection was either completely at random, weighted by typical consumption of F&V in the US or weighted by flavanol content in F&V. Fruits and vegetables sampling was done either with replacement (each F&V item can be selected more than once) or without replacement (reflecting the general recommendation of consuming different types of fruits and vegetables). Values shown are mean and 95% confidence interval of 10 000 simulations. Portion sizes for each F&V were based on 21 CFR § 101.12. At each iteration, flavanol content of a selected food item was drawn from a uniform distribution defined by the reported minimum and maximum concentrations in phenol-explorer.

The majority of study participants, even those with high fruit and vegetable intake, did not achieve a flavanol intake of at least 500 mg day^−1^. To further evaluate these results but using a non-biomarker approach, we simulated the likelihood of ingesting 500 mg day^−1^ flavanol when consuming different numbers of portions of fruit and vegetable following different selection strategies (Fig. 2). Consistent with our biomarker-based analysis, it is unlikely to reach 500 mg day^−1^ of flavanols when prioritising the intake of five portions of fruits and vegetables commonly consumed in the US (including bananas, apples, tomatoes, grapes, oranges, carrots, *etc*.; see SI Table S4 for further details) or selected at random. When prioritising the consumption of fruits and vegetables with higher flavanol content, the probability of consuming 500 mg day^−1^ of flavanols increased but remained less than 50%.

## Discussion

In this study, we investigated whether following current dietary guidelines as measured by fruit and vegetable intake and aHEI sufficiently reflects flavanol intake of at least 500 mg day^−1^, the amount found in COSMOS to significantly reduce cardiovascular mortality by 27%.^3^ To overcome inherent limitations of self-reported dietary assessments to estimate flavanol intake,^13^ we incorporated biomarker data to establish whether study participants consumed at least 500 mg day^−1^ of flavanols.^23^ Our results show that even among participants who adhere to current recommendations for fruit and vegetable intake and healthy dietary patterns, less than 25% of participants achieved a biomarker-estimated flavanol intake of at least 500 mg day^−1^ (Tables 2 and 4). Further, we found very modest differences in flavanol intake between participants with high *versus* low fruit and vegetable intake (Tables 3 and 5). Thus, these results show that an adherence to current dietary guidelines does not ensure a level of flavanol consumption sufficient to expect beneficial cardiovascular effects as shown in the COSMOS trial^3^ and to meet the recommendations for flavanol intake commissioned by the Academy of Nutrition and Dietetics.^8^

The different iterations of the Dietary Guidelines for Americans were designed to meet dietary reference intake (DRI) for nutrients,^9^ not flavanols. Nevertheless, fruits and vegetables are among the main dietary sources of flavanols in the diet^27,28,34^ and are the focus of dietary guidelines. However, our data counter the assumption that meeting recommendations for fruit and vegetable intake will translate to an intake of flavanols of at least 500 mg day^−1^ based on simulations considering the range of flavanol levels present in fruit and vegetables commonly consumed in the US (Fig. 2). When prioritizing fruits and vegetables with high flavanol content, the probability to achieve such an intake moderately increased. However, increasing flavanol intake should not be achieved at the expense of decreasing the diversity of fruits and vegetables consumed. In this context, it is worth considering that flavanol content varies significantly within individual fruits and vegetables based on plant cultivars and breeds, climate, growing and harvest conditions.^35^ The content of (−)-epicatechin even within the same apple variety can fluctuate more than 10-fold,^27^ suggesting that the number of apples to meet an intake of 80 mg of (−)-epicatechin (which is the amount provided in the flavanol intervention tested in COSMOS) could vary from 2 to 29. Furthermore, procyanidins are flavanols that contribute to the astringency in fruits, that is usually identified as an undesired oral sensory quality that breeders may try to remove.^36^ Precisely, the development of specific dietary recommendations for flavanols may positively influence current agricultural practices and food selection methods to increase and optimize the content of flavanols in different plant foods and create an opportunity for food producers to develop varieties with higher flavanol content.

Flavanol intake in the two studies analyzed, COSMOS in the US and EPIC-Norfolk in the UK, showed that the odds of meeting an intake of 500 mg day^−1^ was similar despite differences in the dietary composition between these two countries and the time these studies were conducted. The levels of the more general biomarker of flavanols intake, gVLM_B_, was similar in both cohorts, while SREM_B_ showed higher levels in EPIC-Norfolk (Table 1 and SI Fig. S1). These results are probably due to the higher intake of tea in the UK (Table 1), which represents one of the main sources of (−)-epicatechin in the UK diet.^37^ Nevertheless, the proportion of participants meeting an intake of 500 mg day^−1^ of flavanols remained relatively low even within the top tea consumers in EPIC-Norfolk (Tables 4 and 5). Given the relatively low intake of tea in COSMOS compared to that in EPIC, tea represents a valuable means to increase flavanol intake in the US. However, this should be done with certain considerations. For instance, dietary guidelines for Americans rather implicate tea as a potential means of incorporating added sugars, sweeteners, cream and caffeine.^9^ In addition, while black tea can contribute to the intake of flavanols such as theaflavins and thearubigins,^28,34^ the contribution of these black tea-specific flavanols to the overall health benefits associated with the intake of the flavanols and procyanidins tested in COSMOS remains to be elucidated.

The key strength of this study is the use of validated biomarkers to objectively assess the intake of flavanols. Unlike self-reported dietary assessment methods, which are susceptible to recall bias and other inaccuracies,^12^ biomarkers provide a more reliable measure. As gVLM_B_ and SREM_B_ have different systemic half-lives,^22^ the combination of these biomarkers can provide dietary information across a longer time window. However, several limitations should still be noted, in particular the inter-individual variability of gVLM_B_. gVLM_B_ is based on metabolites derived from the gut microbiota, resulting in larger interindividual variations for gVLM_B_ compared to SREM_B_,^38^ although the variability is comparable with other microbiome derived biomarkers.^39^ The validation of both biomarkers was conducted in an overall younger population,^14^ and while age does not affect urinary SREM concentrations, it is possible that younger populations have relatively higher gVLM levels than older adults.^38^ These limitations, common in concentration biomarkers,^40^ make it difficult to estimate actual intake and we have therefore used a biomarker-based classification system. The thresholds selected for this study were deliberately chosen to overestimate the number of participants with high intake, which means that our findings represent a best-case scenario and it is likely a larger proportion of the population consuming less than 500 mg day^−1^. Sensitivity analyses (SI Fig. S2) show that the estimated proportion of participants meeting the 500 mg day^−1^ threshold remains well below 50%, even with extreme thresholds. This shows that our findings are robust and that meeting dietary recommendations, especially regarding fruits and vegetables, is unlikely to results in high flavanol intake.

For our analyses, aHEI and Eatwell Guide score were used to assess diet quality as this respectively represents previous iterations of the Dietary Guidelines for Americans and dietary recommendations by Public Health England. While other indexes could have been included in the analysis, simulations of flavanol intake showed that even the selection of five portions of fruits and vegetables high in flavanol did not result in flavanol intake of 500 mg day^−1^ or higher (Fig. 2). Thus, it is expected that other dietary patterns recommended by the Dietary Guidelines for Americans such as the DASH and Mediterranean diet would not yield flavanol intakes of at least 500 mg day^−1^. Another notable strength is the inclusion of two large studies of geographically different origin. It should be noted that COSMOS participants, who enrolled in a long-term randomized clinical trial, tended to have a considerably healthier dietary pattern compared to the general US public,^41^ potentially leading to an overestimation of the amount of fruit and vegetable – and flavanol – consumption. Therefore, an even smaller proportion of older US adults likely meets the 500 mg day^−1^ threshold. Future research is therefore needed to evaluate flavanol intake within representative US and UK populations, to better understand the potential public health impact of increased flavanol consumption.

The results from this study also contribute to the general discussion of whether or not developing dietary reference values for bioactives is warranted.^6^ As fruits and vegetables represent a main source of dietary bioactives like flavanols, it is plausible to expect that generic advice to increase fruit and vegetable consumption may not ensure optimal intake of specific bioactives. Such will be the case for other polyphenolic bioactives like anthocyanidins and flavanones as well as other bioactives such as carotenoids, which similar to flavanols, have a distribution in the diet that can vary across specific fruits and vegetables for its content. Considering the importance of diet in disease prevention and healthy aging, further debates and conversations on the development of DRIs or DRI-like values for bioactives offers stimulating opportunities for nutrition research. Perhaps rather than a direct pathway for inclusion of a quantitative DRI target for bioactives, a nuanced and qualitative approach to emphasizing rich food sources, or the development of foods with higher levels of specific bioactives, will be an indirect pathway to address bioactive intake.

## Conclusion

These results show that adherence to current dietary guidelines is not sufficient to address flavanol intake in amounts shown to significantly reduce the risk of cardiovascular death in the COSMOS trial. Hence, the development of specific dietary reference values for flavanols may still be necessary if aiming to increase the intake of these compounds from the diet and translate the health benefits related to the intake of these compounds to the general public. While the development of DRI-like values for flavanols and other bioactive compounds is not presently prioritized in the United States, other authoritative bodies may be able to advance such efforts in a shorter timeline. In this context, recent efforts to develop a framework for the development of recommended intakes for bioactives^6^ as well as the guidelines for flavanol intake commissioned by the Academy of Nutrition and Dietetics^8^ represent valuable steps towards this goal.

## Author contributions

Designed research: JIO, GGCK, HS; performed research: JIO, GGCK; analysed data: JIO, GGCK; wrote the paper: JIO, GGCK, JWE, FS, HS, HDS, JEM.

## Conflicts of interest

J. I. O. and H. S. are employed by Mars, Incorporated, a company engaged in flavanol research and flavanol-related commercial activities. J. W. E. has received investigator-initiated research support from Haleon and Abbott Nutrition during the conduct of this study. H. D. S. and J. E. M. received investigator-initiated grants from Mars Edge, a segment of Mars, incorporated dedicated to nutrition research and products, for infrastructure support and donation of COSMOS study pills and packaging, and Pfizer Consumer Healthcare for donation of COSMOS study pills and packaging during the conduct of the study. H. D. S. additionally reported receiving investigator-initiated grants from Pure Encapsulations, American Pistachio Growers, and Haleon, and honoraria and/or travel for lectures from the Council for Responsible Nutrition, BASF, Haleon, and NIH during the conduct of the study. G. G. C. K. has received an unrestricted grant from Mars, incorporated. The rest of the authors do not have any conflict of interest to declare.

## Supplementary Material

FO-017-D6FO00867D-s001

## Data Availability

Code is available from: https://gitlab.act.reading.ac.uk/xb901875/flavanol-dq. Data from COSMOS trial and associated documentation will be available to users only under a data-sharing agreement. Details on the availability of the study data to other investigators will be on our study website at: https://cosmostrial.org/. EPIC Norfolk aims to make data and samples as widely available as possible whilst safeguarding the privacy of our participants, protecting confidential data and maintaining the reputations of our studies and participants aims. Information on how to request data from EPIC Norfolk can be found here: https://www.epic-norfolk.org.uk/for-researchers/data-sharing/data-requests/. Supplementary information (SI) is available. See DOI: https://doi.org/10.1039/d6fo00867d.
