# Supplementary material for: Adhering to dietary guidelines does not yield flavanol intake levels associated with beneficial cardiovascular effects
Source: Food Funct. 2026 Jun 8;17(12):5715–25. doi: 10.1039/d6fo00867d (PMC13244133; doi:10.1039/d6fo00867d)
Supplement: FO-017-D6FO00867D-s001 [file FO-017-D6FO00867D-s001.pdf]

Supplementary Information (SI) for Food & Function.
This journal is © The Royal Society of Chemistry 2026

# Supplementary information

Supplemental Table 1: Odd ratio (OR) for meeting a biomarker-estimated flavanol intake of at least 500 mg/d in COSMOS and EPIC Norfolk, comparing sex, age and BMI using logistic regression analysis

| | COSMOS | EPIC Norfolk |
|---|---|---|
| Sex (Male vs Female) | 1.34 (1.16; 1.54) | 1.36 (1.27; 1.45) |
| Age (75 vs 65 years old) | 1.21 (1.06; 1.39) | 0.91 (0.83; 0.99) |
| BMI (30 vs 25 kg/m$^2$) | 0.92 (0.85; 0.98) | 1.12 (1.07; 1.17) |

Supplemental Table 2: Association between markers of diet quality and urinary flavanol in COSMOS (difference in log2 biomarker between bottom and top quartile of respective marker, adjusted by age, sex and BMI; 95% confidence interval and Wald-test).

| | $SREM_B$ | | $gVLM_B$ | |
|---|---|---|---|---|
| | ß (95% CI) | p | ß (95% CI) | p |
| Fruits | 0.09 (0; 0.17) | 0.057 | 0.08 (-0.01; 0.16) | **0.003** |
| Vegetables | -0.04 (-0.12; 0.04) | 0.334 | 0.03 (-0.05; 0.11) | 0.314 |
| Fruits & Vegetables | 0.03 (-0.05; 0.11) | 0.650 | 0.06 (-0.02; 0.14) | 0.056 |
| Tea | 0.26 (0.13; 0.40) | **<0.001** | 0.22 (0.08; 0.35) | **0.006** |
| aHEI | 0.13 (0.03; 0.23) | **0.029** | 0.33 (0.23; 0.43) | **<0.001** |

Supplemental Table 3: Association between markers of diet quality and urinary flavanol in EPIC Norfolk (difference in log2 biomarker between bottom and top quartile of respective marker, adjusted by age, sex and BMI; 95% confidence interval and Wald-test).

| | $SREM_B$ | | $gVLM_B$ | |
|---|---|---|---|---|
| | ß (95% CI) | p | ß (95% CI) | p |
| Fruits | -0.06 (-0.11; -0.02) | 0.0107 | -0.15 (-0.21; -0.1) | <0.001 |
| Vegetables | -0.09 (-0.13; -0.06) | <0.001 | -0.1 (-0.14; -0.05) | <0.001 |
| Fruits & Vegetables | -0.08 (-0.12; -0.05) | <0.001 | -0.14 (-0.19; -0.1) | <0.001 |
| Vitamin C | -0.14 (-0.17; -0.1) | <0.001 | -0.09 (-0.13; -0.04) | <0.001 |
| Tea | 0.61 (0.58; 0.65) | <0.001 | 0.28 (0.23; 0.33) | <0.001 |

Supplemental Table 4: List of twenty most frequency consumed fruits and vegetables in NHANES 2017-2019. Portion size was reported according to 21 CFR § 101.12.

| Food | Frequency (number of mentions in dietary recall) | Portion size (g) |
|---|---|---|
| Banana, raw | 1112 | 140 |
| Apple [Dessert], whole, raw | 852 | 140 |
| Tomato, whole, raw | 705 | 85 |
| Grape [Black] | 463 | 140 |
| Grape [Green] | 463 | 140 |
| Orange [Blond] | 450 | 140 |
| Carrot, raw | 394 | 85 |
| Strawberry, raw | 386 | 140 |
| Cucumber, raw | 368 | 85 |
| Green bean, raw | 290 | 85 |
| Melon | 243 | 140 |
| Avocado, raw | 188 | 50 |
| Peanut | 185 | 30 |
| Almond | 172 | 30 |
| Highbush blueberry, raw | 152 | 140 |
| Pineapple | 149 | 140 |
| Lettuce [Green], raw | 126 | 85 |
| Mango | 108 | 140 |
| Grape, raisin | 101 | 40 |
| Pear, whole | 99 | 140 |

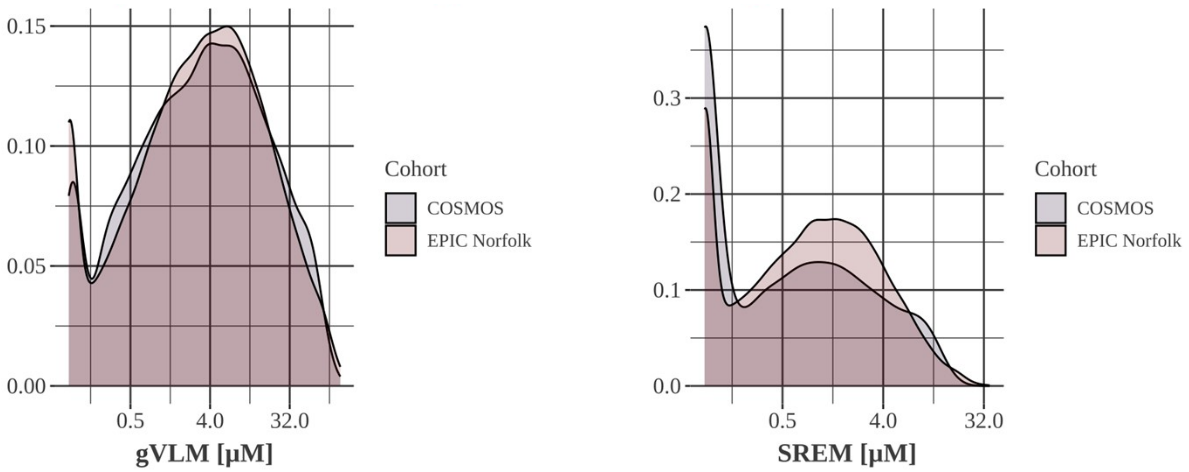


Supplemental Figure 1: Distribution of $gVLM_B$ and $SREM_B$ concentration between COSMOS and EPIC Norfolk. $gVLM_B$: 5-(3′,4′-dihydroxyphenyl)-γ-valerolactone metabolites; $SREM_B$: structurally related (–)-epicatechin metabolites

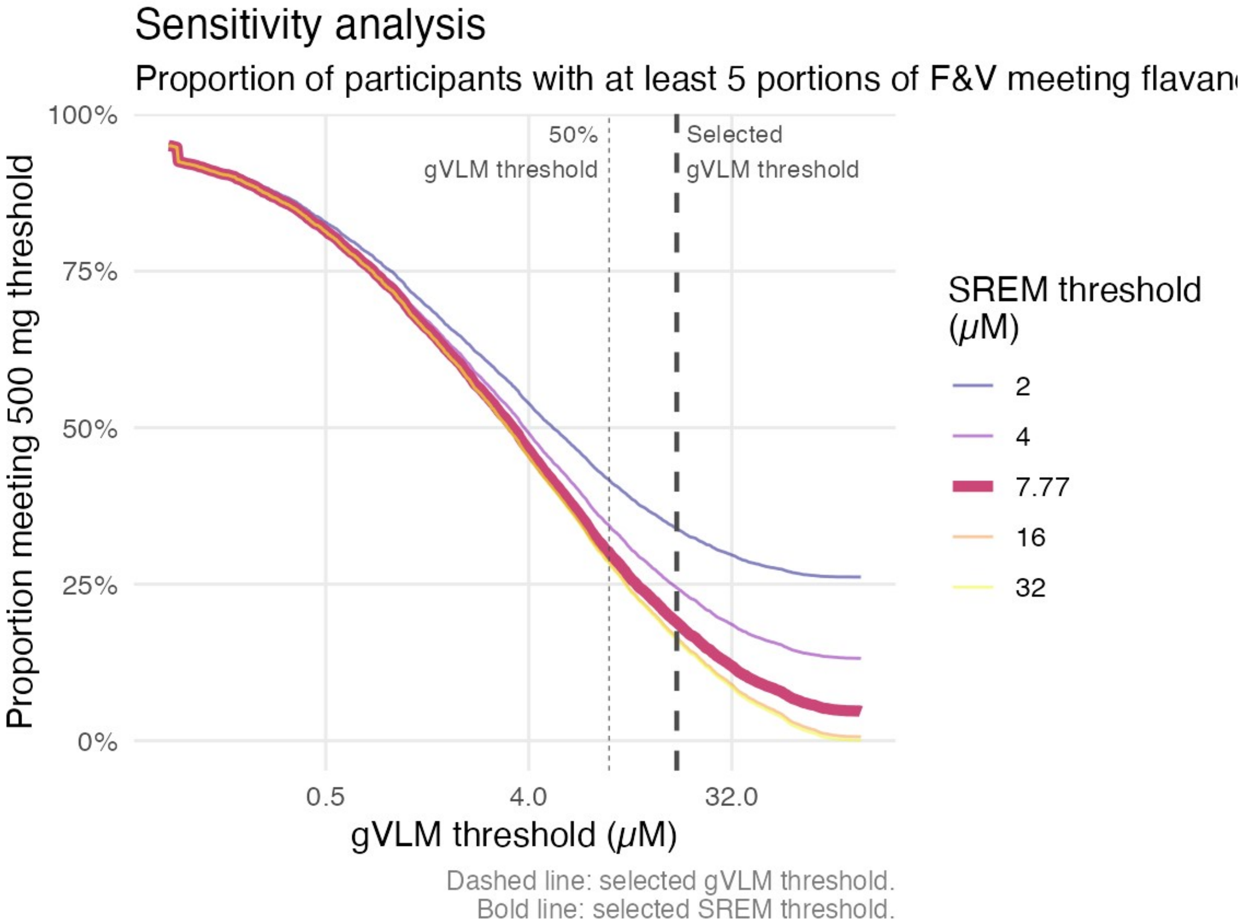

Supplemental Figure 2: Sensitivity analysis assessing changes in the proportion of participants meeting a biomarker-estimated intake of 500 mg of flavanols among participants having at least 5 portions of fruit and vegetables when varying the threshold for the biomarkers 5-(3′,4′-dihydroxyphenyl)-γ-valerolactone metabolites ($gVLM_B$) and structurally related (–)-epicatechin metabolites ($SREM_B$) in COSMOS and EPIC studies.
